# Longitudinal Assessment of Cultural Competencies in Nursing Education: Insights From Students and Nurse Educators

**DOI:** 10.1002/nop2.70339

**Published:** 2025-10-26

**Authors:** Ermira Tartari, Monique Sciortino, Maria Cassar, Laura Visiers‐Jimenez, Maria Isabel Baeza‐Monedero, Josef Trapani, CCA‐EUnurse consortium

**Affiliations:** ^1^ Department of Nursing, Faculty of Health Sciences University of Malta L‐Imsida Malta; ^2^ Department of Statistics & Operations Research, Faculty of Science University of Malta L‐Imsida Malta; ^3^ San Juan de Dios Foundation Madrid Spain; ^4^ San Juan de Dios School of Nursing and Physical Therapy, Health Sciences Department Comillas Pontifical University Madrid Spain

**Keywords:** cultural competence, cultural diversity, intercultural competence, nursing, transcultural competence

## Abstract

**Aim:**

This study aims to evaluate the cultural competencies of undergraduate nursing students and nurse educators and identify associated factors.

**Design:**

Observational, descriptive, longitudinal study conducted in Malta.

**Method:**

Online questionnaires, based on the Cultural Competence Assessment Scale measuring self‐perceived competence and reflections on international experiences, were administered to students during each of the 3 years of an undergraduate nursing programme and once to nurse educators. Descriptive and inferential statistics were applied to examine cultural competence levels and their associations with demographic and experiential factors.

**Results:**

A total of 43, 38 and 34 students participated across the three programme years, respectively, alongside 19 nurse educators (response rates: 51.8% and 70.4%, respectively). Third‐year students demonstrated very good cultural competence with a mean score of 78.24 (out of 100), while educators recorded a slightly lower mean of 73.26. Leisure time abroad before higher education significantly influenced cultural awareness in both first‐ and third‐year students, while age was positively correlated with cultural awareness among final‐year students. For educators, doctoral qualifications were associated with higher cultural awareness, and greater teaching experience correlated with more competent behaviours. Cultural competence improved progressively across the programme, suggesting clinical exposure and maturity play important roles. The inclusion of educators provided a broader perspective, showing that while awareness was strong, behaviour‐based competencies require further support.

**Patient or Public Contribution:**

Developing cultural competence is expected to improve patient care, safety and communication in increasingly diverse healthcare contexts.

## 1 Introduction

Healthcare systems are increasingly challenged by globalisation, migration and multicultural societies, requiring professionals to adapt their practice to diverse populations. The estimated number of persons living in a country other than that of their birth is 281 million; that is approximately 3.6% of the world's population and three times the estimated number 50 years ago (International Organization for Migration (IOM) 2024). These population trends are reshaping healthcare, demanding that the future healthcare workforce develop cultural competence to ensure equitable, safe and person‐centred care (Leininger and McFarland 2002; Flaubert et al. 2021; Shao 2024).

Cultural competence in healthcare refers to the ability to deliver care that respects and responds to patients' cultural backgrounds, values and beliefs (Douglas et al. 2018; Farber 2019). Such competence is crucial for delivering patient‐centred care, reducing healthcare disparities and improving patient outcomes (Abou Hashish et al. 2020; Rahimi et al. 2023).

Leininger's Theory of Diversity and Universality of Care established cultural competence as a cornerstone of nursing, emphasising culturally congruent care through respectful engagement beyond one's own cultural framework (Leininger 1988; Leininger and McFarland 2002). Building on this, Campinha‐Bacote framed cultural competence as an ongoing process of cultural awareness, knowledge acquisition, skill development and the necessity to engage with cultural diversity in healthcare settings (Campinha‐Bacote 2002). Schim and Doorenbos's (2010) three‐dimensional (3D) Model of Cultural Congruence further conceptualised it as the interaction of diversity, awareness, sensitivity and behaviour, highlighting its fluid, context‐dependent, and lifelong nature (Schim and Doorenbos 2010; Raigal‐Aran et al. 2019). In sum, theory informing, guiding and catalysing the evolution of cultural competence in nursing consistently draws upon the contention that the expectation for culture preservation and maintenance, the requirement for reasonable culture accommodation and negotiation, and the need for culture repatterning and restructuring is universally prevalent (McFarland and Wehbe‐Alamah 2019). These three requirements are expected in view of enabling efficient, holistic and effective care toward diverse populations, which characterises most health and social care systems today, and which are predicted for the future.

Cultural competence, and the cultural sensitivity which determines it, is consistently determined as a transient context‐sensitive concept in the literature (Butte and Hristova 2024; Mott Jr. 2003; Podsiadlowski et al. 2013). Its nature underlines the challenges related to establishing and maintaining universal competence across the nurse workforce including the obligation and opportunity of nurse education to address the development of this competence.

Despite the recognised importance of cultural competence, nursing curricula worldwide, particularly within the European Higher Education Area (EHEA), face challenges in effectively integrating cultural competences into undergraduate nursing programmes (Baghdadi and Ismaile 2018). Although the EHEA framework promotes interculturality and includes competencies such as appreciation of diversity, knowledge of other cultures and the ability to work in international contexts (European Parliament and European Council 2013), a gap exists in the practical application of such cultural competencies in nursing education. This gap is amplified by the scarcity of teaching strategies and assessment tools to measure cultural competence development (Caricati et al. 2015; De‐María et al. 2024). In addition, nursing educators often lack adequate training in cultural competence, hindering their ability to teach these competencies effectively (Farber 2019; Kaihlanen et al. 2019; Visiers‐Jiménez et al. 2025). Consequently, nursing students are inadequately prepared to navigate culturally diverse healthcare environments, leading to disparities in the provision of patient care and communication barriers.

### 1.1 Aims

Given the increasing cultural diversity in healthcare settings and the ever‐evolving expectations of nursing practice, this study sought to explore the cultural competencies in nursing students at a university in Malta during their 3‐year undergraduate programme. In addition, the study assessed the cultural competencies of nursing educators to identify gaps in training and inform curriculum development in nurse education.

## 2 Methods

### 2.1 Research Design and Participants

This study adopted an observational, longitudinal and prospective design to examine the development of cultural competencies among undergraduate nursing students enrolled in a three‐year bachelor programme at a university in Malta, as well as nurse educators involved in their education and training. Nursing students who commenced their studies in the year 2021 and were expected to complete their programme by 2024 were eligible to participate. The study used a non‐probability whole population sampling approach. For nurse educators, the inclusion criteria were educators providing theoretical and/or practical education within the same nursing programme. The study was reported in accordance with the STROBE (Strengthening the Reporting of Observational Studies in Epidemiology) guidelines.

### 2.2 Research Instruments

Students' and educators' self‐perceived cultural competence was measured with the Cultural Competence Assessment Scale (CCA), in its original version in English (Doorenbos et al. 2003, 2005). This scale, developed to assess cultural competencies across a broad range of disciplines and educational levels, presented good content validity, acceptable internal consistency (Cronbach's alpha = 0.92) and adequate construct validity (0.40) (Doorenbos et al. 2005). It consists of 25 items divided into two dimensions: Cultural awareness and sensitivity (CAS) (Items 1–11) and cultural competence behaviour (CCB) (Items 12–25). Items are rated on a 7‐point Likert scale, with higher scores indicating greater cultural competence. Participants also have the option to select a ‘no response’ option (Score 0) (refer to Appendices [Link], [Link]).

Two questionnaires were administered to nursing students and nurse educators to capture relevant data targeted to their roles and experiences. The questionnaire for nursing students aimed to assess the evolution of cultural competence throughout their three‐year undergraduate education programme and to examine the influence of internationalisation experiences and has three sections. The first section collected demographic and background information, including age, gender, country of origin, language proficiency and internationalisation experiences, such as participation in Erasmus+ or other internationalisation activities. The second section contained the CAS items, while the third section comprised the CCB items (Doorenbos et al. 2005; Schim and Doorenbos 2010). The questionnaire also assessed the perceived impact of internationalisation experiences and cultural challenges encountered during clinical placements, providing contextual insights into students' cultural adaptation processes and coping strategies in diverse healthcare environments.

The Questionnaire for Nurse Educators (Faculty members) evaluates the cultural competence of nursing educators and its influence on their teaching practices. It comprises three sections. The first section collects demographic and professional background information, including age, gender, educational background, years of teaching experience, language proficiency and participation in internationalisation activities, such as international teaching collaborations. The second section administers the CAS scale to evaluate educators' cultural competence levels. The third section comprises the CCB items, exploring teaching practices and perspectives, investigating how cultural competence influences teaching methods and curriculum design. It also examines perceived challenges in teaching culturally diverse student groups and identifies educators' professional development needs related to cultural competence.

### 2.3 Data Collection

The student questionnaire was administered to the same cohort of students at three critical time points (one at each year of studies) to track the longitudinal development of cultural competence: T1 after completing the first clinical placement, T2 midway through the academic programme and T3 at the end of the final clinical placement. This longitudinal approach facilitates an in‐depth understanding of how cultural competence evolved over time during undergraduate nursing studies. An information meeting was held with a cohort of first‐year nursing students in May 2022, following which the students received a link to the questionnaire, followed by three reminders. The same procedure was repeated when the same cohort of students was in their second and third years of studies.

An invitation to participate and a link to an online version of the Questionnaire for Nurse Educators was sent by email by the research team to all educators involved in providing theoretical and practical education in the undergraduate nursing programme in March 2022.

### 2.4 Ethical Considerations

Participants were provided with an information letter outlining the study's purpose and procedures. In respect of the students' and educators' autonomy, participation was voluntary, and informed consent was inferred through the completion of the online questionnaires. To maintain anonymity, no personally identifiable data were collected. This study received ethical approval on 17th August 2021 from the Faculty of Health Sciences Research Ethics Committee (Reference no: REDACTED).

### 2.5 Statistical Analysis

The scores obtained on the 25 CCA items were summed to yield a total score ranging from 0 – 175. Negatively worded items (Items 1, 2, 5 and 8) were reverse‐scored. Total scores for the CAS and CCB dimensions were also computed. The total scores were then normalised to a scale between 0 and 100 and categorised into four levels: 0–25, > 25–50, > 50–75, and > 75–100, corresponding to low, rather good, good and very good levels of cultural competence, respectively. Descriptive statistics for the normalised and categorised scores were calculated. In addition, statistical tests—including the independent‐samples *t*‐test, Mann–Whitney U test, one‐way ANOVA, Kruskal–Wallis H test, Pearson's product–moment correlation and Spearman's rank‐order correlation—were conducted to identify significant differences based on demographic variables. The choice of statistical test for each demographic variable was based on the type of variable (continuous, categorical with two groups or categorical with three or more groups) and whether the distribution of scores was approximately normal.

## 3 Results

### 3.1 Characteristics of Study Participants

#### 3.1.1 Nursing Students

A total of 43, 38 and 34 nursing students participated during the first, second and third years of the programme, respectively. The majority of students were female (81.4%), with 72.1% of first‐year students in the 18–20 age group. By the third year, the majority of students (64.7%) were in the 21–23 age range (Table 1). Regarding nationality, the majority (86%) were Maltese, while 14% were international students from countries including Italy, Lithuania, Poland, Serbia, the United Kingdom (UK) and Vietnam. A total of 20.9% reported having at least one non‐Maltese parent. Regarding language proficiency, 60.5% reported Maltese as their mother tongue. About half (51.2%) of the students reported proficiency in two or more languages in addition to their mother tongue. Only 9.3% of first‐year students reported previous professional experience in a healthcare context. Regarding internationalisation experiences, only 9.3% of first‐year students had participated in student mobility programmes during higher education, increasing to 58.8% among third‐year students.

**TABLE 1:** Percentage distribution of participants' demographic characteristics.

Demographic variable	Categories	Academics (*n* = 19)	First‐year students (*n* = 43)	Second‐year students (*n* = 38)	Third‐year students (*n* = 34)
Country of origin	Malta	100	86		
	Italy	0	2.3		
	Lithuania	0	2.3		
	Poland	0	2.3		
	Serbia	0	2.3		
	United Kingdom	0	2.3		
	Vietnam	0	2.3		
At least one non‐maltese parent?	Yes	10.5	20.9		
	No	89.5	79.1		
Age	18–20	0	72.1	60.5	8.8
	21–23	0	9.3	26.3	64.7
	24–26	0	14	2.6	8.8
	27–29	0	2.3	2.6	0
	30–39	15.8	0	5.3	8.8
	40–49	42.1	2.3	2.6	8.8
	50–59	15.8	0	0	0
	60+	26.3	0	0	0
Gender	Male	36.8	18.6		
	Female	63.2	81.4		
Religious community	Christian	84.2	72.1		
	Buddhist	0	2.3		
	Muslim	0	2.3		
	Other	0	2.3		
	Do not identify	15.8	18.6		
	Prefer not to say	0	2.3		
Previous professional experience in the healthcare context	Yes	100	9.3		
	No	0	90.7		
Mother tongue	English	15.8	34.9		
	Lithuanian	0	2.3		
	Serbian	0	2.3		
	Maltese	84.2	60.5		
How many languages do you speak in addition to your mother tongue?	1	21.1	46.5		
	2	63.2	41.9		
	3	10.5	9.3		
	4	5.3	0		
Leisure time abroad before higher education	Yes	78.9	60.5		58.8
	No	21.1	39.5		38.2
Study time abroad before higher education	Yes	57.9	14		61.8
	No	42.1	86		38.2
Working time abroad before higher education	Yes	42.1	7		23.5
	No	57.9	93		76.5
Friends from other countries or cultures before higher education	Yes	94.7	74.4		85.3
	No	5.3	25.6		14.7
Erasmus+ experience in higher education	Once	10.5	9.3		58.8
	Twice	10.5	0		0
	Three times	10.5	0		0
	> 3 times	10.5	0		0
	No	57.9	90.7		41.2
Experiences at internationalisation activities ‘At Home’	Yes	84.2	11.6		32.4
	No	15.8	88.4		67.6
Nursing degree	Yes	94.7			
	No	5.3			
Level of education	Postgraduate	47.4			
	Doctorate	52.6			
Employment status	Full‐time	84.2			
	Part‐time	15.8			
Years of experience as a teacher	< 5	21.1			
	5 to < 10	10.5			
	10 to < 15	10.5			
	15 to < 20	10.5			
	20 to < 25	21.1			
	25+	26.3			
Years of experience as a healthcare provider	< 5	5.3			
	5 to < 10	15.8			
	10 to < 15	42.1			
	15 to < 20	10.5			
	20 to < 25	5.3			
	25+	21.1			

*Note:* Empty cells in this table indicate that the corresponding question was not asked for the respective group.

#### 3.1.2 Nursing Educators

A total of 19 out of the 27 eligible nurse educators participated in the study (response rate: 70.4%). A majority of 63.2% were females and 36.8% males. All educators were 30 years or older, with 42.1% in the 40–49 age group and 15.8% in both the 30–39 and 50–59 age groups.

Regarding academic qualifications, 94.7% held a nursing degree, with 47.4% having postgraduate education and 52.6% holding doctoral qualifications. The majority (84.2%) were in full‐time employment. Teaching experience varied, with 47.4% having 20 or more years of experience and 21.1% having less than 5 years. All educators (100%) reported previous professional experience in healthcare settings. The average healthcare provider experience was predominantly in the 10–15 year range (42.1%). In terms of language proficiency, a majority of 84.2% reported Maltese as their first language, with 78.9% speaking at least two languages in addition to their mother tongue. Regarding internationalisation experiences, 42.1% of educators reported participation in teaching or training mobility programmes. A high proportion of nurse educators (84.2%) reported also participating in ‘internationalisation at home’ activities. Table 1 summarises the demographic characteristics of the participants, categorised by group (academics, first‐year, second‐year and third‐year students).

### 3.2 CCA Items' Reliability and Response Distributions

In this study, Cronbach's alpha values for the CAS and CCB items were 0.516 and 0.897, respectively. While the CCB dimension demonstrated high internal consistency, the CAS dimension showed lower reliability and may warrant further examination.

#### 3.2.1 Nursing Students' Responses to CAS Items

Students' responses to cultural awareness items showed generally high levels of cultural awareness with some variations across the years. A large majority of students across all year groups agreed or strongly agreed with the statement, ‘I believe that everyone should be treated with respect no matter what their cultural heritage’ (first‐year: 95.4%; second‐year: 97.4% and third‐year: 97.1%) (Table 2). Recognition of cultural influences on healthcare concepts increased across years, with the statement ‘I understand that people from different cultures may define the concept of “healthcare” in different ways’ receiving agreement from 76.7% of first‐year, 81.6% of second‐year, and 94.1% of third‐year students. Understanding cultural diversity in healthcare was perceived as important, with 79.1% of first‐year, 94.7% of second‐year, and 97.1% of third‐year students agreeing to: ‘Many aspects of culture influence health and healthcare’. Rejection of cultural stereotypes showed improvement across the years. For the statement ‘Language barriers are the only difficulties for recent immigrants’, disagreement increased from 58.1% of first‐year to 70.6% of third‐year students. Similarly, for the statement ‘People with a common cultural background think and act alike’, disagreement increased from 16.3% of first‐year to 26.5% of third‐year students (Table 2).

**TABLE 2:** Reported frequencies (%) of students and educators for the cultural awareness and sensitivity (CAS) items.

Statement	Academics (*n* = 19)					First‐year students (*n* = 43)					Second‐year students (*n* = 38)					Third‐year students (*n* = 34)				
	A	SA	N	SD	D	A	SA	N	SD	D	A	SA	N	SD	D	A	SA	N	SD	D
Race is the most important factor in determining a person's culture.	15.8	15.8	15.8	10.5	42.1	11.6	16.3	16.3	11.6	44.2	18.4	29.0	10.5	7.9	34.2	8.8	8.8	17.7	5.9	58.8
People with a common cultural background think and act alike.	21.1	15.8	0.0	10.5	52.6	20.9	23.3	18.6	16.3	20.9	29.0	23.7	10.5	13.2	23.7	5.9	38.2	5.9	26.5	23.5
Many aspects of culture influence health and healthcare.	73.7	15.8	0.0	0.0	10.5	53.5	25.6	9.3	7.0	4.7	71.1	23.7	2.6	0.0	2.6	64.7	32.4	2.9	0.0	0.0
Aspects of cultural diversity need to be assessed for each individual, group, and organisation.	89.5	5.3	5.3	0.0	0.0	65.1	14.0	14.0	2.3	4.7	71.1	7.9	18.4	0.0	2.6	76.5	14.7	8.8	0.0	0.0
If I know about a person's culture, I do not need to assess their personal preferences for health services.	10.5	0.0	0.0	5.3	84.2	2.3	0.0	14.0	4.7	79.1	7.9	0.0	10.5	10.5	71.1	0.0	2.9	5.9	8.8	82.4
Spirituality and religious beliefs are important aspects of many cultural groups.	68.4	31.6	0.0	0.0	0.0	81.4	7.0	7.0	4.7	0.0	89.5	7.9	2.6	0.0	0.0	82.4	8.8	5.9	0.0	2.9
Individuals may identify with more than one cultural group.	79.0	15.8	5.3	0.0	0.0	72.1	14.0	9.3	2.3	2.3	86.8	2.6	10.5	0.0	0.0	79.4	17.7	2.9	0.0	0.0
Language barriers are the only difficulties for recent immigrants.	5.3	5.3	0.0	5.3	84.2	20.9	4.7	7.0	9.3	58.1	18.4	15.8	7.9	10.5	47.4	5.9	8.8	5.9	8.8	70.6
I believe that everyone should be treated with respect no matter what their cultural heritage.	100.0	0.0	0.0	0.0	0.0	95.4	0.0	4.7	0.0	0.0	97.4	2.6	0.0	0.0	0.0	97.1	0.0	0.0	0.0	2.9
I understand that people from different cultures may define the concept of ‘healthcare’ in different ways.	100.0	0.0	0.0	0.0	0.0	76.7	9.3	9.3	0.0	4.7	81.6	13.2	2.6	2.6	0.0	94.1	5.9	0.0	0.0	0.0
I think that knowing about different cultural groups helps direct my work with individuals, families, groups, and organisation.	94.7	5.3	0.0	0.0	0.0	86.1	7.0	7.0	0.0	0.0	92.1	2.6	5.3	0.0	0.0	94.1	5.9	0.0	0.0	0.0

Abbreviations: A, strongly agree/agree; D, disagree/strongly disagree; N, neutral/no opinion; SA, somewhat agree; SD, somewhat disagree.

#### 3.2.2 Nursing Students' Responses to CCB Items

Students' self‐reported cultural behaviours showed generally positive patterns with improvement across the years. The behaviour ‘I avoid using generalizations to stereotype groups of people’ was reported as always/very often by 74.4% of first‐year and 79.4% of third‐year students. Actively seeking cultural information improved over time and was reported as always/very often by 41.9% of first‐year and 52.9% of third‐year students. Documentation practices varied across year groups, with only 32.6% of first‐year and 41.2% of third‐year students reporting that they always/very often document cultural assessments (Table 3).

**TABLE 3:** Reported frequencies (%) of students and educators for the cultural competence behaviour (CCB) items.

Statement	Academics (*n* = 19)					First‐year students (*n* = 43)					Second‐year students (*n* = 38)					Third‐year students (*n* = 34)				
	VO	O	FT	N	NS	VO	O	FT	N	NS	VO	O	FT	N	NS	VO	O	FT	N	NS
I include cultural assessment when I do patient/individual or collective evaluation.	15.8	36.8	5.3	26.3	15.8	41.9	16.3	16.3	23.3	2.3	39.5	21.1	23.7	10.5	5.3	44.1	14.7	14.7	20.6	5.9
I seek information on cultural needs when I identify new patients and families/people in my practice.	42.1	5.3	26.3	21.1	5.3	41.9	14.0	23.3	16.3	4.7	44.7	21.1	18.4	7.9	7.9	52.9	11.8	14.7	11.8	8.8
I have resource webpages, books available to help me learn about patients and families/people from different cultures.	15.8	26.3	0.0	26.3	31.6	30.2	11.6	9.3	16.3	32.6	31.6	10.5	15.8	15.8	26.3	35.3	17.7	11.8	17.7	17.7
I use a variety of sources to learn about the cultural heritage of other people.	36.8	15.8	0.0	26.3	21.1	27.9	11.6	20.9	20.9	18.6	31.6	15.8	21.1	10.5	21.1	50.0	8.8	14.7	17.7	8.8
I ask patients and families/people to tell me about their explanations of health and illness.	26.3	10.5	31.6	10.5	21.1	44.2	7.0	9.3	23.3	16.3	39.5	23.7	7.9	15.8	13.2	41.2	20.6	11.8	11.8	14.7
I ask patients and families/people to tell me about their expectations for care.	26.3	21.1	21.1	15.8	15.8	37.2	14.0	0.0	27.9	20.9	44.7	15.8	13.2	18.4	7.9	32.4	23.5	14.7	14.7	14.7
I avoid using generalisations to stereotype groups of people.	63.2	15.8	10.5	5.3	5.3	74.4	11.6	9.3	2.3	2.3	65.8	13.2	15.8	2.6	2.6	79.4	5.9	5.9	8.8	0.0
I recognise potential barriers to services/education that might be encountered by different people.	63.2	15.8	21.1	0.0	0.0	58.1	14.0	18.6	7.0	2.3	52.6	26.3	13.2	7.9	0.0	64.7	23.5	8.8	2.9	0.0
I act to remove obstacles for people of different cultures when I identify such obstacles.	68.4	15.8	15.8	0.0	0.0	60.5	18.6	14.0	2.3	4.7	63.2	18.4	10.5	7.9	0.0	61.8	17.7	17.7	2.9	0.0
I act to remove obstacles for people of different cultures when patients and families/others identify such obstacles to me.	68.4	15.8	15.8	0.0	0.0	58.1	16.3	18.6	4.7	2.3	63.2	23.7	7.9	5.3	0.0	70.6	14.7	8.8	5.9	0.0
I welcome feedback from patients/students about how I relate to others with different culture.	84.2	5.3	5.3	5.3	0.0	74.4	7.0	7.0	4.7	7.0	65.8	15.8	10.5	0.0	7.9	79.4	8.8	5.9	2.9	2.9
I find ways to adapt my services/work to patient and family/individual and collective cultural preferences.	73.7	10.5	0.0	10.5	5.3	67.4	11.6	4.7	9.3	7.0	68.4	13.2	15.8	2.6	0.0	61.8	26.5	8.8	2.9	0.0
I document cultural assessments.	15.8	5.3	5.3	21.1	52.6	32.6	4.7	23.3	11.6	27.9	29.0	15.8	23.7	13.2	18.4	41.2	14.7	29.4	8.8	5.9
I document the adaptations I make with patients and families/students.	15.8	5.3	5.3	15.8	57.9	32.6	7.0	25.6	9.3	25.6	47.4	7.9	21.1	13.2	10.5	47.1	11.8	29.4	2.9	8.8

Abbreviations: FT, sometimes/few times; *N*, never; NS, not sure; O, somewhat often/often; VO, always/very often.

#### 3.2.3 Nursing Educators' Responses to CAS Items

All educators (100%) agreed/strongly agreed with the statement ‘I believe that everyone should be treated with respect no matter what their cultural heritage’ and ‘I understand that people from different cultures may define the concept of healthcare in different ways’ (Table 2). Strong recognition of cultural influences on healthcare was evident, with 89.5% of educators agreeing that ‘Aspects of cultural diversity need to be assessed for each individual, group and organisation’ and 73.7% agreeing that ‘Many aspects of culture influence health and healthcare’. Rejection of cultural stereotypes was also strong, with 84.2% of educators disagreeing with the statements ‘Language barriers are the only difficulties for recent immigrants’ and ‘If I know about a person's culture, I do not need to assess their personal preference for health services’ (Table 2).

#### 3.2.4 Nursing Educators' Responses to CCB Items

A total of 63.2% of educators reported always/very often avoiding generalisations to stereotype groups of people, and the same proportion indicated regularly recognising potential barriers to services that different individuals might encounter. Behaviours related to modifying services to accommodate cultural diversity were reported frequently, with 73.7% of educators reporting always/very often finding ways to adapt their work to cultural preferences. Also, 84.2% reported always/very often welcoming feedback about how they relate to others with different cultures. Documentation practices were less commonly reported, with only 15.8% of educators reporting always/very often documenting cultural assessments and cultural adaptation they make (Table 3).

### 3.3 Cultural Competence Assessment

Table 4 presents descriptive statistics for the CCA, CAS and CCB normalised scores, as well as percentage frequencies for the corresponding categorised normalised scores across all participant groups. Categories 0–25, > 25–50, > 50–75 and > 75–100 correspond to low, rather good, good and very good levels of cultural competence, respectively.

**TABLE 4:** Descriptive and percentage frequency statistics of the CCA, CAS and CCB scores.

Score statistics	Academics (*n* = 19)	First‐year students (*n* = 43)	Second‐year students (*n* = 38)	Third‐year students (*n* = 34)
*Cultural competence assessment (CCA)*				
Mean	73.26	73.48	75.19	78.24
Standard deviation	9.76	11.87	10.72	11.54
Minimum	48.57	44.57	49.71	52.00
Lower quartile	68.00	68.00	68.14	69.57
Median	74.29	73.14	74.00	80.00
Upper quartile	80.00	82.29	82.57	87.57
Maximum	90.86	94.86	96.57	96.57
Frequency for *low level of competen*ce (< = 25)	0	0	0	0
Frequency for *rather good level of competence* (> 25–50)	5.3	4.7	2.6	0
Frequency for *good level of competence* (> 50–75)	57.9	53.5	50	32.4
Frequency for *very good level of competence* (> 75–100)	36.8	41.9	47.4	67.6
*Cultural awareness and sensitivity (CAS)*				
Mean	84.89	78.95	79.63	84.34
Standard deviation	5.81	8.51	8.90	8.05
Minimum	75.32	54.55	63.64	68.83
Lower quartile	80.52	75.32	71.43	77.60
Median	85.71	77.92	80.52	85.06
Upper quartile	88.31	85.71	86.69	88.96
Maximum	96.10	94.81	96.10	98.70
Frequency for *low level of competen*ce (< = 25)	0	0	0	0
Frequency for *rather good level of competence* (> 25–50)	0	0	0	0
Frequency for *good level of competence* (> 50–75)	0	23.3	34.2	11.8
Frequency for *very good level of competence* (> 75–100)	100	76.7	65.8	88.2
*Cultural competence behaviour (CCB)*				
Mean	64.12	69.17	71.70	73.44
Standard deviation	15.17	17.75	16.13	18.52
Minimum	25.51	26.53	33.67	28.57
Lower quartile	56.12	62.24	60.71	59.44
Median	64.29	68.37	71.94	76.53
Upper quartile	75.51	80.61	84.95	89.29
Maximum	89.80	100	100	100
Frequency for *low level of competen*ce (< = 25)	0	0	0	0
Frequency for *rather good level of competence* (> 25–50)	15.8	14	7.9	14.7
Frequency for *good level of competence* (> 50–75)	57.9	48.8	50	29.4
Frequency for *very good level of competence* (> 75–100)	26.3	37.2	42.1	55.9

#### 3.3.1 Nursing Students' Overall Cultural Competence (CCA)

The mean CCA scores showed a progressive increase across the 3 years of the nursing degree programme: 73.48 (SD = 11.87) for first‐year, 75.19 (SD = 10.72) for second‐year and 78.24 (SD = 11.54) for third‐year students, representing a 4.76‐point increase. In third‐year, the majority of the students (67.6%) demonstrated a very good level of competence, while the remaining 32.4% demonstrated a good level of competence (Table 4).

#### 3.3.2 Nursing Students' Overall Cultural Awareness and Sensitivity (CAS)

The CAS dimension showed improvement over the 3 years, with mean scores of 78.95 (SD = 8.51) for first‐year, 79.63 (SD = 8.90) for second‐year, and 84.34 (SD = 8.05) for third‐year students. No student in any of the 3 years demonstrated low or rather good levels of cultural awareness and sensitivity (Table 4).

#### 3.3.3 Nursing Students' Overall Cultural Competence Behaviour (CCB)

The CCB dimension showed an increase in mean scores across the programme: 69.17 (SD = 17.75) for first‐year, 71.70 (SD = 16.13) for second‐year, and 73.44 (SD = 18.52) for third‐year students. No student in any year demonstrated a low level of cultural competence behaviour. The percentage with a very good level increased steadily: 37.2% in first‐year, 42.1% in second‐year, and 55.9% in third‐year students (Table 4).

#### 3.3.4 Nursing Educators' Overall Cultural Competence (CCA)

Nurse educators showed a mean CCA score of 73.26 (SD = 9.76). The minimum score was 48.57 and the maximum was 90.86. None of the educators demonstrated a low level of competence, 5.3% a rather good level, while 57.9% showed a good level and 36.8% a very good level of competence (Table 4).

#### 3.3.5 Nursing Educators' Overall Cultural Awareness and Sensitivity (CAS)

In the CAS dimension, educators showed a mean score of 84.89 (SD = 5.81). The minimum score was 75.32, and the maximum was 96.10. All educators (100%) demonstrated a very good level of cultural awareness and sensitivity, with none in the lower categories (Table 4).

#### 3.3.6 Nursing Educators' Overall Cultural Competence Behaviour (CCB)

In the CCB dimension, educators had a mean score of 64.12 (SD = 15.17). The minimum score was 25.51, and the maximum was 89.80. None demonstrated a low level of cultural competence behaviour, while 15.8% showed a rather good, 57.9% a good and 26.3% a very good level (Table 4).

### 3.4 Statistical Analysis of Scores by Demographic Characteristics

Tables 5, 6, 7 present the inferential statistics for the CCA, CAS and CCB scores, respectively, by demographic variables across all participant groups.

**TABLE 5:** Statistical test results of cultural competence assessment (CCA) by demographic characteristics.

Demographic variable	CCA result	Academics (*n* = 19)	First‐year students (*n* = 43)	Second‐year students (*n* = 38)	Third‐year students (*n* = 34)
At least one non‐maltese parent?	Test statistic	17.000^b^	0.174^a^		
	*p*	1.000	0.863		
Age	Test statistic	0.368^e^	0.131^f^	0.035^f^	0.287^f^
	*p*	0.121	0.403	0.836	0.100
Gender	Test statistic	0.367^a^	−1.149^a^		
	*p*	0.718	0.257		
Religious community *(christian/other/do not identify)*	Test statistic	3.875^c^	0.178^c^		
	*p*	0.066	0.837		
Previous professional experience in the healthcare context	Test statistic	N/A (one group)	−1.150^a^		
	*p*		0.257		
How many languages do you speak in addition to your mother tongue? *(only one/more than one)*	Test statistic	0.787^a^	−1.186^a^		
	*p*	0.442	0.243		
Leisure time abroad before higher education	Test statistic	−0.294^a^	1.686^a^		0.814^a^
	*p*	0.772	0.099		0.422
Study time abroad before higher education	Test statistic	0.827^a^	1.427^a^		156.500^b^
	*p*	0.420	0.161		0.484
Working time abroad before higher education	Test statistic	0.461^a^	0.292^a^		1.036^a^
	*p*	0.651	0.771		0.308
Friends from other countries or cultures before higher education	Test statistic	6.000^b^	−1.229^a^		84.000^b^
	*p*	0.737	0.226		0.603
Erasmus+ experience in higher education *(once/multiple times/no)*	Test statistic	0.358^d^	0.636^c^		0.002^c^
	*p*	0.836	0.430		0.963
Experiences at internationalisation activities ‘At Home’	Test statistic	0.382^a^	−1.860^a^		−0.648^a^
	*p*	0.707	0.070		0.521
Level of education	Test statistic	−1.468^a^			
	*p*	0.160			
Years of experience as a teacher	Test statistic	0.390^e^			
	*p*	0.099			
Years of experience as a healthcare provider	Test statistic	−0.041^f^			
	*p*	0.867			

^a^ Independent‐samples *t*‐test.

^b^ Mann–Whitney U test.

^c^ One‐way ANOVA.

^d^ Kruskal–Wallis H test.

^e^ Pearson's product–moment correlation.

^f^ Spearman's rank‐order correlation.

#### 3.4.1 Nursing Students

Among nursing students, differences in both the CCA and CCB scores based on demographic variables were not statistically significant at the 0.05 level across any year of the programme (Tables 5 and 7: all *p*‐values > 0.05).

Age demonstrates a statistically significant moderate positive correlation with the CAS scores among third‐year students (ρ = 0.482, *p* = 0.004), suggesting that older third‐year students demonstrate higher cultural awareness and sensitivity (Table 6). Moreover, leisure time abroad before higher education significantly impacts the CAS scores in first‐year students (*t* = 2.381, *p* = 0.022) (Table 6). First‐year students who had spent leisure time abroad demonstrated significantly higher CAS scores (mean = 81.32, SD = 6.99) compared to those without such experience (mean = 75.32, SD = 9.52). Similarly, leisure time abroad significantly impacts the CAS scores among third‐year students (*t* = 2.860, *p* = 0.008) (Table 6). Third‐year students with leisure travel experience demonstrated significantly higher CAS scores (mean = 87.60, SD = 7.57) than their counterparts without this experience (mean = 80.52, SD = 5.83).

**TABLE 6:** Statistical test results of cultural awareness and sensitivity (CAS) by demographic characteristics.

Demographic variable	CAS result	Academics (*n* = 19)	First‐year students (*n* = 43)	Second‐year students (*n* = 38)	Third‐year students (*n* = 34)
At least one non‐maltese parent?	Test statistic	16.500^b^	0.220^a^		
	*p*	0.947	0.827		
Age	Test statistic	0.015^e^	0.014^f^	0.136^f^	0.482^f^
	*p*	0.952	0.929	0.416	**0.004**
Gender	Test statistic	−0.163^a^	−0.733^a^		
	*p*	0.872	0.468		
Religious community (christian/other/do not identify)	Test statistic	1.703^c^	1.461^d^		
	*p*	0.209	0.482		
Previous professional experience in the healthcare context	Test statistic	N/A (one group)	−0.809^a^		
	*p*		0.476		
How many languages do you speak in addition to your mother tongue? *(only one/more than one)*	Test statistic	0.944^a^	0.180^a^		
	*p*	0.359	0.858		
Leisure time abroad before higher education	Test statistic	0.180^a^	2.381^a^		2.860^a^
	*p*	0.859	**0.022**		**0.008**
Study time abroad before higher education	Test statistic	1.261^a^	0.820^a^		−0.324^a^
	*p*	0.224	0.417		0.748
Working time abroad before higher education	Test statistic	0.618^a^	1.349^a^		0.159^a^
	*p*	0.545	0.185		0.875
Friends from other countries or cultures before higher education	Test statistic	12.500^b^	−0.333^a^		1.073^a^
	*p*	0.632	0.741		0.291
Erasmus+ experience in higher education *(once/multiple times/no)*	Test statistic	2.055^d^	0.389^c^		0.363^c^
	*p*	0.358	0.536		0.551
Experiences at internationalisation activities ‘At Home’	Test statistic	0.708^a^	−0.646^a^		−1.027^a^
	*p*	0.489	0.522		0.312
Level of education	Test statistic	−3.001^a^			
	*p*	**0.008**			
Years of experience as a teacher	Test statistic	−0.086^e^			
	*p*	0.726			
Years of experience as a healthcare provider	Test statistic	−0.077^f^			
	*p*	0.753			

*Note:* Bold *p*‐values indicate statistical significance (*p* < 0.05).

^a^ Independent‐samples *t*‐test.

^b^ Mann–Whitney U test.

^c^ One‐way ANOVA.

^d^ Kruskal–Wallis H test.

^e^ Pearson's product–moment correlation.

^f^ Spearman's rank‐order correlation.

#### 3.4.2 Nursing Educators

Among nursing educators, differences in the CCA scores based on demographic variables were not statistically significant at the 0.05 level (see Table 5: all *p*‐values > 0.05).

Academic qualification level was, however, significantly associated with the CAS scores (*t* = −3.001, *p* = 0.008) (Table 6). Educators with doctoral qualifications demonstrated significantly higher cultural awareness and sensitivity (mean = 88.05, SD = 5.54) compared to those with postgraduate qualifications (mean = 81.39, SD = 3.90).

Years of teaching experience demonstrated a statistically significant moderate positive correlation with the CCB scores (*r* = 0.474, *p* = 0.040) (Table 7). This suggests that educators with greater teaching longevity tend to show increased culturally competent behaviours in their professional practice.

**TABLE 7:** Statistical test results of cultural competence behaviour (CCB) by demographic characteristics.

Demographic variable	CCB result	Academics (*n* = 19)	First‐year students (*n* = 43)	Second‐year students (*n* = 38)	Third‐year students (*n* = 34)
At least one non‐maltese parent?	Test statistic	17.000^b^	0.125^a^		
	*p*	1.000	0.901		
Age	Test statistic	0.418^e^	0.153^f^	−0.021^f^	0.188^f^
	*p*	0.075	0.329	0.901	0.288
Gender	Test statistic	0.473^a^	−0.774^a^		
	*p*	0.643	0.461		
Religious community (christian/other/do not identify)	Test statistic	3.318^c^	0.264^c^		
	*p*	0.086	0.769		
Previous professional experience in the healthcare context	Test statistic	N/A (one group)	−0.786^a^		
	*p*		0.436		
How many languages do you speak in addition to your mother tongue? *(only one/more than one)*	Test statistic	0.618^a^	−1.481^a^		
	*p*	0.544	0.147		
Leisure time abroad before higher education	Test statistic	−0.393^a^	1.123^a^		141.000^b^
	*p*	0.699	0.268		0.703
Study time abroad before higher education	Test statistic	0.574^a^	1.388^a^		162.500^b^
	*p*	0.573	0.173		0.362
Working time abroad before higher education	Test statistic	0.343^a^	−0.149^a^		1.101^a^
	*p*	0.736	0.882		0.279
Friends from other countries or cultures before higher education	Test statistic	6.000^b^	−1.345^a^		74.500^b^
	*p*	0.737	0.186		0.925
Erasmus+ experience in higher education *(once/multiple times/no)*	Test statistic	1.327^d^	0.512^c^		0.066^c^
	*p*	0.515	0.478		0.799
Experiences at internationalisation activities ‘At Home’	Test statistic	0.228^a^	−1.979^a^		−0.372^a^
	*p*	0.823	0.055		0.712
Level of education	Test statistic	−0.877^a^			
	*p*	0.393			
Years of experience as a teacher	Test statistic	0.474^e^			
	*p*	**0.040**			
Years of experience as a healthcare provider	Test statistic	−0.029^f^			
	*p*	0.905			

*Note:* Bold *p*‐values indicate statistical significance (*p* < 0.05).

^a^ Independent‐samples *t*‐test.

^b^ Mann–Whitney U test.

^c^ One‐way ANOVA.

^d^ Kruskal–Wallis H test.

^e^ Pearson's product–moment correlation.

^f^ Spearman's rank‐order correlation.

## 4 Discussion

This study sought to explore the cultural competencies of a cohort of undergraduate nursing students and their educators at a university in Malta and to identify factors associated with these competencies. The response rates obtained in this survey were satisfactory, with 70.4% of academics and 51.8% of first‐year students participating, which compares favourably to similar surveys assessing cultural competencies in nursing education (Abou Hashish et al. 2020; Antón‐Solanas et al. 2021; Ličen et al. 2021; Osmancevic et al. 2023; Cruz et al. 2018; Repo et al. 2017; Shepherd et al. 2019), and which is substantially higher than the mean response rate for online surveys in published research (Menon and Muraleedharan 2020; Wu et al. 2022). The demographic distribution in the samples of both students and educators reflects those in the respective entire cohorts reasonably well, thus enhancing the confidence with which the findings may be generalised within the context of this university and those with a similar structure.

The notable increase in the proportion of students who had participated in student mobility programmes between the first and final years of studies could be attributed to multiple factors. While this might suggest that the nursing programme successfully encouraged participation in such experiences over time, this pattern could also reflect selective retention, whereby students interested in international experiences (and potentially the more culturally aware ones) were more likely to remain in the study while less interested ones dropped out. Conversely, it may suggest that previous participation in such mobility enhanced the interest in the topic under investigation, and, therefore, in cultural competence. Indeed, in a previous mixed‐methods evaluation of study‐abroad experiences of students from an earlier cohort of the same undergraduate programme, 89% of the participants perceived that their student mobility had enhanced their intercultural awareness to a very large or considerable extent, with ‘exposure to nursing beyond the national shores’ and ‘context‐sensitivity of nursing care delivery’ emerging as prominent themes (Trapani and Cassar 2020).

Given the minimal formal education and training specifically on cultural competence in the curriculum, the significant improvement in cultural awareness and culturally sensitive behaviour across the years of study may be attributed to the students' personal development and maturity, and improved awareness gained through the clinical placements which, invariably, led them to be exposed to healthcare providers and service users from diverse backgrounds and settings. The latter explanation is supported by the rapid rise in the proportion of persons from diverse backgrounds and settings in Malta, particularly in the last decade (Borg 2025; National Statistics Office [Malta] 2024). However, there are a number of caveats to be considered. First, the data are based on self‐reporting, which may introduce response bias. Second, as students' progress toward becoming fully fledged nurses, they may be more prone to social desirability bias by selecting responses that align with professional expectations from a healthcare professional. Third, the sample may have suffered from self‐selection bias, with participants who are more likely to be culturally competent being also more likely to maintain their participation throughout the study. Nonetheless, the consistent improvement across nearly all statements and behaviours is encouraging.

While the scores for overall cultural competence and its subscales among third‐year students showed a marked increase from the previous years of the programme, it is particularly interesting to note that the students' scores were at least as good as, and in the case of the cultural competence behaviour, substantially better than those of educators. Cultural Awareness and Sensitivity scores of first‐year students started off as lower than those of academics but reached a similar level by the third year. This pattern may be interpreted in several ways. First, the generally younger generation of students may have been exposed to greater cultural diversity in both their personal and their student/professional lives. Second, the demographic characteristics indicate that the student cohort constituted a more culturally diverse group in terms of country of origin, having at least one parent from a different country of origin and identifying with non‐Christian backgrounds. A counter argument, however, is that a higher proportion of educators reported experiences of travelling for leisure/work and having friends from different countries compared to students. Third, the fact that by their final year of studies students had essentially increased their CAS scores to the level of academics may reflect students' proximity to graduation and professional status; thus, achieving almost similar scores may be a reflection of the fact that they were now ‘almost nurses’.

For culturally competent behaviour, the mean score of final year nursing students was substantially higher than that of nurse educators. While this may appear surprising, it likely reflects the different nature of nursing students' and nurse academics' professional lives. At the university in which this study was conducted, in conformity with EU directives (European Parliament and European Council 2013), students spend approximately half their hours of study in a variety of clinical placements and, therefore, in close contact with healthcare professionals and service users, several of whom are from diverse backgrounds. Conversely, educators generally spend most of their time engaged in teaching, research and academic administration. Indeed, a closer examination of the constituents of this subscale reveals that approximately three‐quarters of the statements are related to culturally appropriate behaviour in several aspects of patient/family assessment, care provision or service delivery—activities which nursing students, but not nurse educators, regularly engage in several times a week, and potentially explains the substantially higher scores achieved by nursing students.

Similar to the recent results reported by Visiers‐Jiménez et al. (2025), in this study both students and educators scored higher in cultural awareness and sensitivity than in cultural competence behaviour. This may be considered an example of the well‐established gap between theory and practice (Saifan et al. 2021; Singh et al. 2024; Tambunan 2024) and affirms the challenges educators face in terms of imparting culturally competent and sensitive practice (Abubakari et al. 2024; Osmancevic et al. 2023; Paric et al. 2021).

The positive significant association between cultural competence behaviour scores and academics' years of teaching experience may indicate that educators may develop greater cultural competence throughout their careers, possibly due to increased exposure over time. The significantly higher degree of cultural awareness and sensitivity of educators with doctoral degrees is potentially attributable to extended periods of travel and broader exposure associated with doctoral studies, especially since doctoral nursing programmes in Malta were only introduced relatively recently. Cicolini et al. (2015) similarly attribute this influence to the greater exposure to cultural diversity that occurs in higher education. Likewise, Visiers‐Jiménez et al. (2025), in their study of nursing faculty from 17 European countries, found that nursing faculty with higher educational attainment exhibit greater cultural competence. Osmancevic et al. (2023) also reported an association between higher educational levels and higher levels of cultural competence.

Among students, the significant association between age and CAS score affirms the improvement in cultural awareness and sensitivity acquired as students' progress in their undergraduate programme, a finding that is in accordance with previous research (Cruz et al. 2018; Reyes et al. 2013). The similar positive association with previous leisure travel is congruent with earlier work among a comparable cohort that found improved perceived cultural awareness among Maltese nursing students who participated in ERASMUS+ mobility for studies, but which often included substantial leisure travel (Trapani and Cassar 2020).

### 4.1 Study Strengths and Limitations

The study has several methodological strengths. The longitudinal design allowed for tracking changes in cultural competence over time within the same cohort of students, providing valuable insights into development trajectories throughout a nursing programme. The inclusion of both students and educators offered a comprehensive view of cultural competence across different stakeholders in nursing education. In addition, the use of a validated CCA assessment tool with established psychometric properties enhanced the reliability of the findings.

However, some limitations must be acknowledged. First, the self‐reporting nature of the questionnaires may have introduced socially desirable bias, with participants potentially responding in ways they perceived as favourable rather than reporting their actual behaviours and attitudes. Second, the decreasing sample size across the 3 years suggests possible self‐selection bias, with culturally interested students potentially more likely to continue participation.

Due to the single‐institution design, generalisability to other educational, geographic, temporal or environmental contexts requires caution. Furthermore, the small sample sizes in demographic subgroups reduced statistical power, making it more difficult to detect significant differences and increasing the likelihood of Type II errors (Serdar et al. 2021). While the study captures longitudinal changes, it cannot definitively attribute these to specific educational or experiential components without controlling for confounding variables.

The Cultural Competence Assessment tool, while validated, may not capture all dimensions of cultural competence relevant to the Maltese healthcare context, particularly because cultural competence is inherently transient, ephemeral, and context‐dependent (Butte and Hristova 2024; Mott Jr. 2003; Podsiadlowski et al. 2013). Despite these limitations, the findings are likely to have resonance and relevance for similar nursing education contexts.

### 4.2 Recommendations for Nursing Education and Practice

The study findings highlight actionable recommendations for improving cultural competence development in nursing education. First, nursing curricula should systematically integrate cultural competence training throughout the programme, moving beyond isolated modules to embedded approaches that connect theory with clinical practice. The progressive improvement in cultural competence scores across the 3 years of study, despite minimal formal training, suggests that clinical placements and experiential learning play a crucial role in developing cultural competence (Antón‐Solanas et al. 2021; Chang et al. 2019; Liu and Li 2023; Powell 2020). Clinical experiences should, therefore, be designed to expose students to diverse populations and settings rather than assuming cultural competence will develop incidentally.

Second, structured faculty development programmes should be implemented to enhance educators' cultural competence, addressing the identified gap between educators' awareness and behavioural competence. The identified gap between cultural awareness and culturally competent behaviour indicates a need for nursing curricula to move beyond theoretical knowledge toward practical application. Curricula should specifically address the decision‐making and actions associated with Leininger's (1988) three modes of nursing decisions and actions, which underpin transcultural theory and cultural competence: culture preservation and maintenance; culture accommodation and negotiation; and culture repatterning and restructuring. Guided by this triad of foci, innovative teaching methods focused on translating awareness into behaviour are required to bridge this gap.

Third, institutions should strategically create, facilitate and fund international mobility experiences and exchanges for both students and educators, recognising their value in developing cultural awareness and sensitivity. The transient and context‐sensitive nature of cultural competence calls for periodic, targeted educational and experiential opportunities which enable the development of the awareness and sensitivity required for competence development (Farber 2019).

Fourth, institutions should implement faculty development initiatives focused on cultural competence, ensuring educators are supported toward developing and maintaining competence to effectively model and impart these competencies to students (Abou Hashish et al. 2020; Rahimi et al. 2023).

Fifth, partnerships with demographically diverse healthcare facilities should be established to ensure students gain exposure to varied cultural contexts throughout their educational journey. Evidence‐based reflective practice tools should be incorporated to help students critically analyse and synthesise learning from these cross‐cultural experiences.

Mindful of the challenges and risks associated with narrowly applying the concept of cultural competence, educators and policymakers should consciously safeguard against reinforcing institutional gaps in cultural competence. In congruence with the literature (Gustafson 2005; Haqawi et al. 2024; Wesp et al. 2018), initiatives should enable effective and efficient culture preservation, accommodation and repatterning consistent with optimal care delivery, rather than simply highlighting diversity in a population.

Finally, the significantly better scores in cultural competence behaviours and overall cultural awareness among third‐year students demonstrate the cumulative effect of nursing education on students' competence development. This finding points to the potential value of peer education approaches, where senior students could contribute to enhancing junior students' cultural competence. The literature (Stone et al. 2013; Zhang et al. 2022) reports that such peer involvement, which already takes place informally in some clinical placements, is perceived as valuable by both nursing students (Eraydın and Güven 2024) and preceptors (Jassim et al. 2022). Given the identified gap in nurse educators' competence, structured peer education initiatives may serve as an important complementary strategy in developing future nurses' cultural competence.

### 4.3 Recommendations for Further Research

Future research should explore the impact of specific educational interventions on cultural competence development. Mixed‐methods approaches are essential to triangulate self‐reported data with objective measures and indicators of cultural competence in clinical practice, addressing the methodological limitations identified in this study (Ahmed et al. 2018; The Lewin Group 2002).

Research examining service users' perspectives on nurses' cultural competence would add valuable dimensions to our understanding of this construct's practical implications. Additionally, multi‐site comparative studies across institutions and countries could identify evidence‐based practices for developing cultural competence, enabling context‐sensitive adaptations while identifying universal principles. Future investigations should also evaluate the intersection of cultural competence with other dimensions of nursing practice, including ethical decision‐making, patient safety and healthcare outcomes in increasingly diverse societies.

## 5 Conclusion

This longitudinal study offers valuable insights into cultural competence development among nursing students and educators within the Maltese healthcare education context. The findings demonstrate a progressive increase in cultural competence throughout the undergraduate nursing programme, with third‐year students attaining very good levels of cultural competence, surpassing those of educators on the same programme. The observed improvements in both cultural awareness sensitivity and in culturally competent behaviours suggest that the synergistic combination of clinical experiences, developmental maturation, and exposure to diverse populations contributes substantially to cultural competence acquisition.

The study identifies several influential factors in cultural competence development. International leisure experiences significantly influenced cultural awareness scores, highlighting the potential value of cross‐cultural immersion beyond formal educational contexts. Age demonstrated a positive correlation with cultural awareness, specifically among third‐year students, suggesting that maturity may facilitate deeper cultural understanding. Among nurse educators, academic qualification showed significant associations with cultural awareness, while teaching experience correlated with culturally competent behaviours, underscoring the multifaceted nature of professional development in this domain.

These findings have implications for nursing education curriculum design, professional development initiatives and policy formulation. By highlighting the development trajectory of cultural competence and identifying influential factors, it provides an empirical foundation for preparing culturally responsive healthcare practitioners for increasingly diverse healthcare environments. Future research should build upon these insights by examining specific pedagogical interventions that most effectively bridge the gap between cultural awareness and culturally competent behaviours in clinical practice.

## Author Contributions

Conceptualisation: E.T., J.T. and M.C.; methodology: L.V.‐J. and M.I.B.‐M.; data curation and formal analysis: M.S.; validation: E.T., J.T., M.C., M.S. M.I.B.‐M. and L.V.‐J.; writing original draft: E.T., J.T., M.C. and M.S.; writing, review and editing: all authors have contributed, read and agreed to the final version of the manuscript.

## Conflicts of Interest

The authors declare no conflicts of interest.

## Supporting information


**Appendix S1:** CCA‐EUnurse (1st Year): Cultural Competence Assessment Questionnaire for first‐year nursing students, establishing baseline cultural competence measures at the beginning of nursing education.

**Appendix S2:** CCA‐EUnurse (2nd Year): Cultural Competence Assessment Questionnaire for second‐year nursing students, assessing progression of cultural competence at the midpoint of nursing education.

**Appendix S3:** CCA‐EUnurse (3rd Year): Cultural Competence Assessment Questionnaire for third‐year nursing students, evaluating cultural competence development in the final year of the nursing degree programme.

**Appendix S4:** CCA‐EUnurse (Teachers): Cultural Competence Assessment Questionnaire for nurse educators at European universities, measuring cultural awareness, sensitivity and behaviours in health teaching contexts.

## Data Availability

The data that support the findings of this study are available from the corresponding author upon reasonable request.
