# Supplementary material for: Longitudinal Assessment of Cultural Competencies in Nursing Education: Insights From Students and Nurse Educators
Source: Nurs Open. 2025 Oct 26;12(10):e70339. doi: 10.1002/nop2.70339 (PMC12554084; doi:10.1002/nop2.70339)
Supplement: Supplementary file 2 — Appendix S2: CCA‐EUnurse (2nd Year): Cultural Competence Assessment Questionnaire for second‐year nursing students, assessing progression of cultural competence at the midpoint of nursing education. [file NOP2-12-e70339-s002.pdf]

CCA_EUnurse EN 2º

The following questionnaire is addressed to NURSING STUDENTS at European universities. The questions in this questionnaire aim to collect information about how you think, feel and act with others in the context of health care and health service environments and in academic settings. Please try to answer all questions. If you are not sure or do not have an opinion on any of the questions, please use the options "no opinion" or "not sure". There are no "right" or "wrong" answers. Completing this questionnaire is VOLUNTARY. It will take about 10 minutes. We will contact you once more at the end of your training. To do this, we will ask you to specify in this questionnaire the same contact email address that you indicated in the first questionnaire, that you regularly use and that you will keep until the end of your training. The data in this questionnaire are ANONYMOUS and will only be used for research purposes. This study complies with current regulations regarding anonymised data processing. If you have any questions, you can contact the principal investigator of the study at any time: Laura Visiers Jiménez (Centro Universitario San Rafael-Nebrija, Madrid, Spain): lvisiers@nebrija.es

AGE (YEARS) __________________________________

EUROPEAN COUNTRY WHERE YOU ARE STUDYING

◯ Belgium
◯ Denmark
◯ France
◯ Germany
◯ Greece
◯ Holand
◯ Ireland
◯ Italy
◯ Latvia
◯ Lithuania
◯ Malta
◯ Portugal
◯ Scotland
◯ Slovakia
◯ Sweden
◯ Switzerland
◯ Turkey

HIGHER EDUCATION INSTITUTION	◯ Belgium:Thomas More University of Applied Sciences -Campus Lier (TML)<br>◯ Belgium:Thomas More University of Applied Sciences -Campus Mechelen (TMM)<br>◯ Belgium:Thomas More University of Applied Sciences -Campus Turnhout (TMT)<br>◯ Denmark: Oslo Metropolitan University. Faculty of Health Science (OMU)<br>◯ Denmark:University College Absalon (UCA)<br>◯ Denmark:University College Copenhaguen (KP) ◯ France:Institut de Formation en Soins Infirmiers du CHRU de Nancy (IFSI)<br>◯ Germany:Frankfurt University of Applied Sciences (FRA-UAS) ◯ Greece:University of Thessaly (UTH) ◯ Holand: Hanzehogeschool Groningen (HUAS) ◯ Ireland:University College Cork (UCC) ◯ Italy:University of Verona. Campus Bolzano (UVB)<br>◯ Italy:University of Verona (UV)<br>◯ Italy:Centro Studi San Giovanni di Dio, Roma (Centro Studi FBF) ◯ Italy:Humanitas (HUNIMED) ◯ Latvia:Rīgas Stradiņa universitāte (RSU) ◯ Lithuania:Vilniaus Kolegija/University of Applied Sciences (VIKO)<br>◯ Malta:University of Malta (UoM)<br>◯ Portugal: Escola Superior de Enfermagem de Lisboa (ESEL) ◯ Portugal: Escola Superior de Enfermagem do Porto (ESENF)(ESENF)<br>◯ Portugal: Escola Superior de Enfermagem San José de Cluny (ESESJCluny)<br>◯ Portugal: Escola Superior de Saúde Fernando Pessoa: Porto (ESS-FP) ◯ Portugal: Universidade de Aveiro (UA)<br>◯ Scotland: Glasgow Caledonian University (GCU) ◯ Slovakia: Slovak Medical University in Bratislava (SMU) ◯ Sweden: Linnaeus University (LU) ◯ Switzerland: Bern University of Applied Sciences (BFH)<br>◯ Turkey: Ege University Izmir (EUI)
CURRENT YEAR OF THE EDUCATION	◯ 1º ◯ 2º ◯ 3º ◯ 4º

CULTURAL COMPETENCE ASSESSMENT ORIGINAL VERSION (CCA)

Please, select how are you feeling about the following statements:

		Strongly agree	Agree	Somewhat agree	Neutral	Somewhat disagree	Disagree	Strongly disagree	No opinion
1	The race is the most important factor in determining a person's culture.	○	○	○	○	○	○	○	○
2	People with a common cultural background think and act alike.	○	○	○	○	○	○	○	○
3	Many aspects of culture influence health and healthcare.	○	○	○	○	○	○	○	○
4	Aspects of cultural diversity need to be assessed for each individual, group, and organization.	○	○	○	○	○	○	○	○
5	If I know about a person's culture, I do not need to assess their personal preferences for health services.	○	○	○	○	○	○	○	○
6	Spirituality and religious beliefs are important aspects of many cultural groups.	○	○	○	○	○	○	○	○
7	Individuals may identify with more than one cultural group.	○	○	○	○	○	○	○	○
8	Language barriers are the only difficulties for recent immigrants.	○	○	○	○	○	○	○	○
9	I believe that everyone should be treated with respect no matter what their cultural heritage.	○	○	○	○	○	○	○	○
10	I understand that people from different cultures may define the concept of "healthcare" in different ways.	○	○	○	○	○	○	○	○
11	I think that knowing about different cultural groups helps direct my work with individuals, families, groups, and organization.	○	○	○	○	○	○	○	○

CULTURAL COMPETENCE ASSESSMENT ORIGINAL VERSION (CCA)

Please, select how often you perform each of the following activities:

		Always	Very often	Somewhat often	Often	Sometimes	Few times	Never	Not sure
12	I include cultural assessment when I do patient or collective evaluation.	○	○	○	○	○	○	○	○
13	I seek information on cultural needs when I identify new patients and families in my practice.	○	○	○	○	○	○	○	○
14	I have resource webpages, books and other materials available to help me learn about patients and families from different cultures.	○	○	○	○	○	○	○	○
15	I use a variety of sources to learn about the cultural heritage of other people.	○	○	○	○	○	○	○	○
16	I ask patients and families to tell me about their explanations of health and illness.	○	○	○	○	○	○	○	○
17	I ask patients and families to tell me about their expectations for care.	○	○	○	○	○	○	○	○
18	I avoid using generalizations to stereotype groups of people.	○	○	○	○	○	○	○	○
19	I recognize potential barriers to services that might be encountered by different people.	○	○	○	○	○	○	○	○
20	I act to remove obstacles for people of different cultures when I identify such obstacles.	○	○	○	○	○	○	○	○
21	I act to remove obstacles for people of different cultures when patients and families identify such obstacles to me.	○	○	○	○	○	○	○	○
22	I welcome feedback from patients about how I relate to others with different culture.	○	○	○	○	○	○	○	○
23	I find ways to adapt my services to patient and family cultural preferences.	○	○	○	○	○	○	○	○

24

	I document cultural assessments.	○	○	○	○	○	○	○	○
25	I document the adaptations I make with patients and families.	○	○	○	○	○	○	○	○

Contact email address that will allow to track your future answers while maintaining your anonymity.

____
